# The safety and efficacy of ultrasound-guided erector spinae plane block in postoperative analgesic of PCNL: A systematic review and meta-analysis

**DOI:** 10.1371/journal.pone.0288781

**Published:** 2023-07-14

**Authors:** Jiang Liu, Shirong Fang, Yuxi Wang, Lin Wang, Lunan Gao, Tingting Xin, Yuxiu Liu

**Affiliations:** 1 School of Nursing, Weifang Medical University, Weifang, China; 2 Weifang People’s Hospital, Weifang Medical University, Weifang, China; AOU Policlinico ’Rodolico - San Marco’, ITALY

## Abstract

**Background:**

The patients received percutaneous nephrolithotomy (PCNL) with severe postoperative pain and discomfort. The erector spinae plane block (ESPB), as a new anesthesia method of plane block, has a positive effect on postoperative analgesia. But evidence of ESPB in PCNL is still lacking. The objective of this study was to systematically analyze the postoperative analgesic effect of ESPB in patients receiving PCNL.

**Methods:**

The literature searching was conducted in PubMed, EMBASE, Cochrane Library and Clinical Trial Database (clinicaltrials.gov). Two independent researchers screened the included studies and extracted data. Meta-analysis was conducted by using the random-effect model with 95% confidence intervals. Chi-squared test with a significance level of 0.1 was utilized to evaluate the heterogeneity of included studies. The subgroup analysis and meta-regression analysis were conducted in studies with high heterogeneity. The publication bias was assessed based on whether there were discrepancies between prospective trial registration and reported protocols.

**Results:**

There were 8 studies involving 456 patients assessing the efficacy of ESPB in reducing postoperative pain score of PCNL compared with no block or other blocks, such as subcutaneous infiltration, general anesthesia or TPVB intrathecal morphine. ESPB was a significantly effective and safe anesthesia method, which not only improved postoperative pain response (MD −1.76; 95% CI −2.57 to −0.94; I ^2^ = 85%; p<0.01), but also reduced analgesic consumption (MD −16.92; 95% CI −26.25 to −7.59; I ^2^ = 92.2%; p<0.01) and prolonged the time of first request for postoperative analgesia (MD 93.27; 95% CI 35.79 to 150.75; I ^2^ = 85.3%; p = 0.001) in patients receiving PCNL without significant postoperative complications (MD 0.80; 95% CI 0.31 to 2.03; I ^2^ = 0%; p = 0.404).

**Conclusions:**

Compared with no block or other blocks, the ESPB was a safe and effective anesthesia for patients receiving PCNL.

## Introduction

Acute postoperative pain is one of the most common postoperative complications. Studies reports that more than 80% of patients suffer from postoperative pain. However, less than 50% of patients get effective pain relief [1]. According to the findings of recent studies, the incidence of patients experiencing moderate or severe pain in the first 24 hours after surgery was still up to 60%. Moreover, this proportion has remained mostly unchanged over the past 30 years [2]. Effective postoperative analgesia is crucial to improve patient comfort, facilitate early mobilization, and promote an efficient recovery [3]. The objective of our review was to analyze the postoperative analgesic effect of ESPB compared with no block or other blocks in adult patients undergoing PCNL. Moreover, we also reported the safety of ESPB for PCNL.

Percutaneous nephrolithotomy (PCNL), as a widely used minimally invasive surgical technique, is the gold standard surgical method for treatment of patients with large and complicated renal stone. Nevertheless, the PCNL can result in severe postoperative pain and discomfort due to the influence factors such as dilatation of the renal capsule, parenchymal canal, peritubal distension and pressure of the nephrostomy tube [4–6]. If there is no effective control and intervention, pain related to PCNL may cause a series of adverse acute and chronic complications including anxiety, nausea, ventilatory dysfunction, increased consumption of myocardial oxygen, immune function injury and myocardial injury [7, 8]. This not only increases the discomforts postoperative recovery of patients, but also prolongs hospital stay and even leads to the incidence of persistent postoperative pain [9].

The controversy still existed in the ideal method of anesthesia for PCNL. Presently, the common methods of anesthesia for PCNL include general anesthesia and regional anesthesia such as spinal anesthesia (SA) and epidural anesthesia (EA) [10]. Previously, research shows that the postoperative analgesia based on opioid has many opioid-related side effects such as itchyskin, vomiting, nausea and dizziness. However, the discomfort caused by opioid-related side effects is usually more than the pain itself. According to current evidence, epidural analgesia is no longer considered as the “gold standard”. In recent years, a new anesthesia method of plane block named erector spinae plane block has received attention after firstly introduced by Forero et al. in 2016 [11]. It has gained more and more attention from patients, clinicians and researchers due to its simple, safety, significant analgesic effect and small postoperative side reactions. Moreover, many studies also reports that the ESPB had a significant effect on postoperative analgesia in PCNL [12–14].

As an interfacial block method, ESPB exerts the block effect of dorsal and ventral spinal nerves by injecting a local anesthetic into the deep surface of the erector spinae muscle and the surface of the transverse process. Anesthetics gradually spread to the peripheral paravertebral and intercostal areas to exert effects. ESPB can significantly avoid the injury of peripheral nerve and blood vessel, and effectively reduce the difficulty of blocking surgery. Currently, studies have confirmed the significant analgesic effect of ESPB after chest, abdomen and spine surgery [15, 16]. But the application of ESPB in PCNL is still lacking high-quality evidence. Although many RCTs have studied the analgesic effect of ESPB for PCNL in recent years, the results are controversial. Therefore, a high-quality meta-analysis is needed to comprehensively analyze the postoperative analgesic efficacy and safety of this relatively new technique on PCNL.

## Methods

This systematic review and meta-analysis followed the Preferred Reporting Items for Systematic Reviews and Meta-Analyses (PRISMA) guidelines. The protocol of this meta-analysis was registered on PROSPERO (CRD42022380570).

### Search strategy

Two reviewers independently searched PubMed, EMBASE, Cochrane Library and ClinicalTrials.gov from inception to December 1, 2022. The search strategy used terms related to erector spinae plane block, including "erector spinae plane", "erector spinae plane block", etc. The language of literature search was limited to English. Grey literature and reference lists were also screened to identify relevant studies.

### Inclusion and exclusion criteria

Inclusion criteria: (1) all randomized controlled trials investigating the analgesic effect of ESPB in patients received PCNL, (2) all kinds of control interventions such as no block or other block, (3) population older than 18 yrs. Excluded criteria: (1) study outcomes incomplete or unreported, (2) animal study, (3) researches with overlapping data.

### Articles selection and data extraction

All searched literature were managed in EndNote© X9. After eliminating duplicates, the retrieved studies were screened by two reviewers independently according to titles and abstracts. Then, the full-text examination of the included articles was conducted by another two investigators independently to assess appropriateness to be included in our study. Any disagreement was solved through mutual discussion. Data extraction was performed by separate reviewers utilizing the self-designed table and pilot tested with three literatures. The primary outcome was to compare the postoperative pain score (VAS, NRS) between the ESPB group and control group. The secondary outcomes included analgesic consumption, the time to first rescue analgesia, the incidence of PONV and all kinds of postoperative complications related to ESPB.

### Bias assessment

The quality of individual study was assessed by two separate investigators using the Cochrane Risk-of-Bias Tool [17]. The bias-risk of each study was classified as low risk, high risk, or unclear risk of bias according to the six types of bias items. The disagreement was solved by discussing with the third researcher. The funnel plot was not utilized to test the asymmetry since fewer than ten studies were included in meta-analysis and the size of the studies was similar [18]. Additionally, in order to avoid the impact of selective reporting risk and to provide the highest quality evidence, this study greatly emphasizes the prospective trial registration of the included RCTs. Selective reporting risk was assessed based on whether there were discrepancies between registered and reported protocols.

### Statistical analysis

The comparison of continuous variables including pain score, analgesic consumption and the time to first rescue analgesia was done by weighted mean difference (WMD). As for comparative results for dichotomous variables, such as the incidence of PONV, were compared with relative risk. If the results were expressed in terms of median and interquartile range, transformation mean and SD were done by using the formula described by Hozo et al [19]. If there were two or more studies reported the same or similar results using the same tools, the random-effect model with 95% confidence intervals (CIs) was utilized to perform the meta-analysis. Chi-squared test with a significance level of 0.1 was utilized to evaluate the heterogeneity of included studies. We divided the heterogeneity into low heterogeneity (*I*^*2*^<50%) and significant heterogeneity (*I*^*2*^>50%) according to the classification described by PRISMA-P [20]. The sensitivity analysis was conducted in studies with high heterogeneity by using the means of removing individual studies one by one to observe whether the heterogeneity was decreased. The reason of heterogeneity generating was explored by using subgroup analysis and meta-regression analysis. Considering that pain sensitivity decreases in people over 50 yrs, a subgroup analysis was conducted based on the mean age of 50 yrs." All statistical analysis of pooled data were performed in STATA 16.0.

## Results

### Characteristics of the included studies

**Fig 1:**
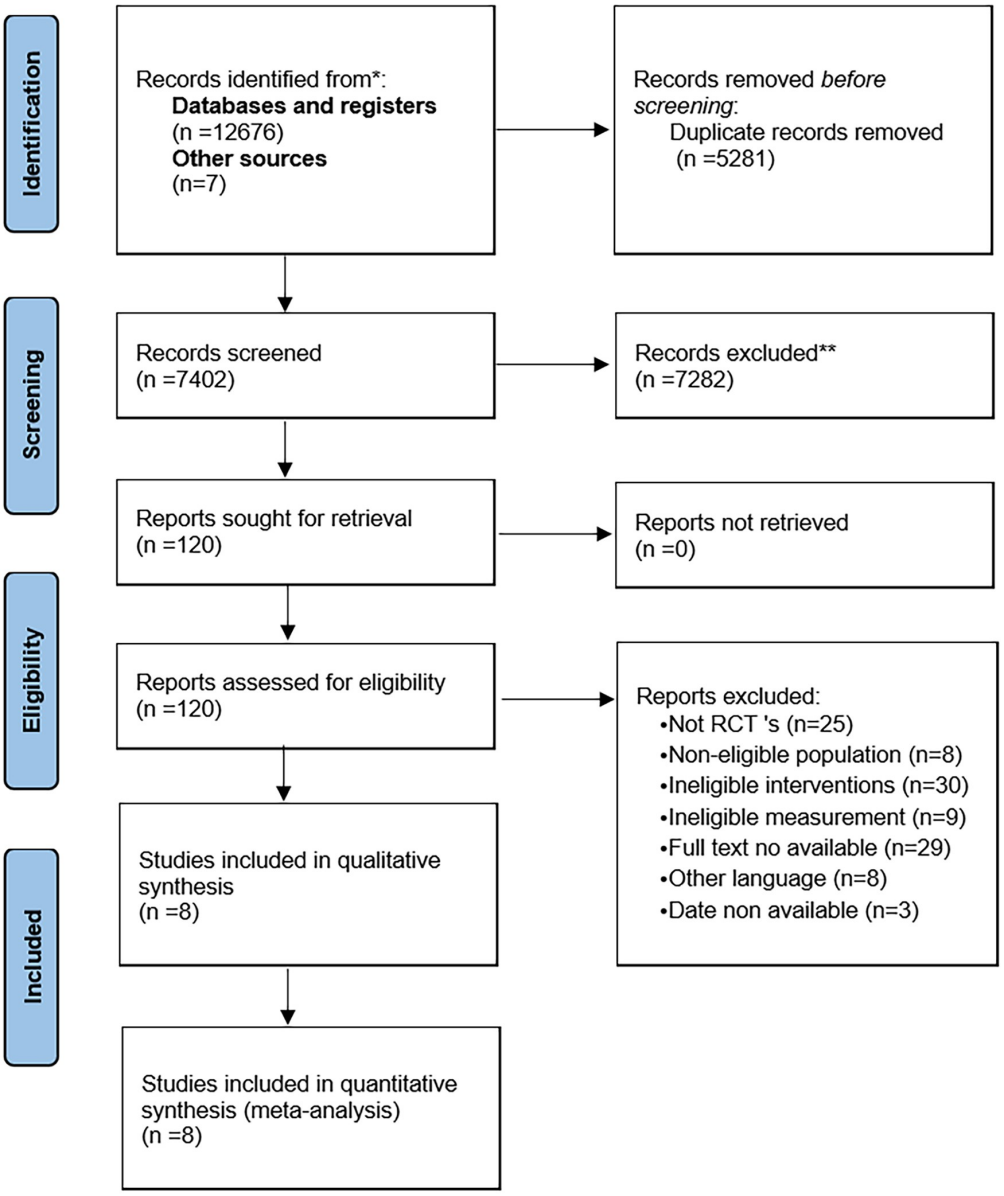
PRISMA Flow diagram of this meta-analysis.

A total of 12,683 studies were identified. After removing duplicates and screening titles/abstracts, 120 full-text articles were assessed for eligibility. Eight studies with 456 participants were included in the meta-analysis. The study selection process is shown in Fig 1.

All 8 randomized controlled trials were prospectively registered (S1 Table). They were published from 2019 to 2022 and conducted in different countries [12, 21–27]. One was carried out in Turkey [12, 23], four in India [21, 22, 26, 27], one in Egypt [24], and one in Poland [25]. The mean age of patients was >50 years in 3 studies [12, 23, 25], and ≤50 years in 5 studies [21, 22, 24, 26, 27]. A total of five studies analyzed ESPB versus no block [12, 21, 23, 24, 26], one study compared ESPB with general anesthesia [25], one study compared ESPB with TPVB intrathecal morphine [27], and one study compared ESPB with subcutaneous infiltration [22]. The included studies used different tools to assess postoperative pain, but all were standard and reliable. Postoperative pain was assessed on the VAS scale in six studies [12, 21, 23, 25–27], with the remaining two using the NRS scale [22, 24]. Additionally, six studies performed the ESPB using bupivacaine [12, 21–25], with the remaining two using ropivacaine [26, 27]. There was also difference in the types of postoperative analgesics included: tramadol was used in 4 studies for postoperative analgesia [12, 22, 23, 26], tramadol combined with paracetamol in one study [21], fentanyl in one study [27], morphine in one study [24], and nalbuphine in one study [25]. The general information of all the studies was summarized in S1 Table.

### Evaluation of evidence quality

**Fig 2:**
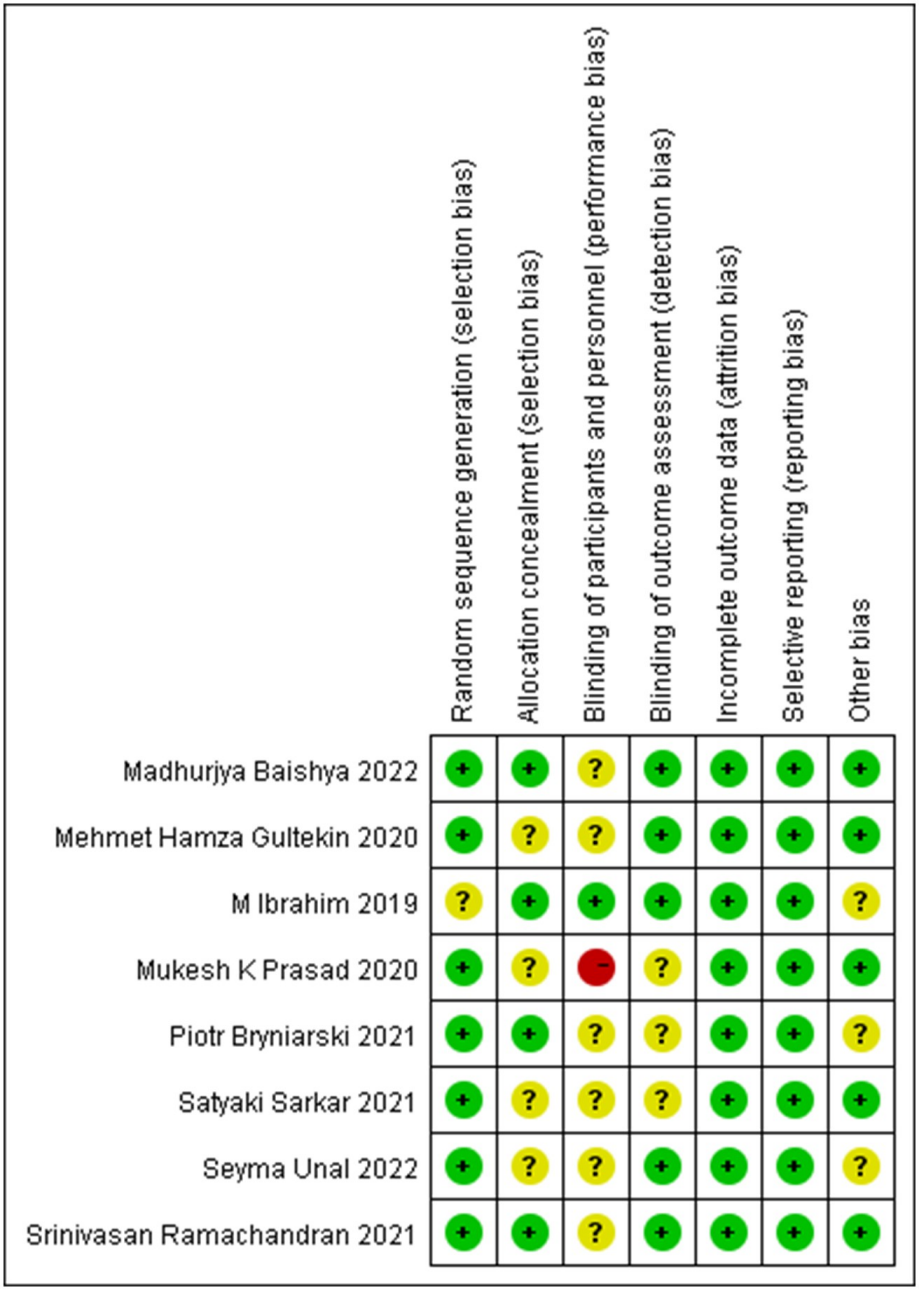
Risk of bias summary.

All studies described the method of randomization details, but one was defined as unclear risk of bias due to insufficient information [24]. Only four studies reported the method of allocation concealment [22, 24, 25, 27], and others did not specify in detail the process used. Considering the nature of the interventions, it was difficult to ensure blindness to participants and personnel, and all studies were unclear risk of bias, except one was low-risk [24] and one was high-risk [26]. Three studies was defined as unclear risk of bias because of insufficient information about blinding for outcome assessment [21, 25, 26], and the remaining studies were all low-risk. There were no studies existing the high-risk of attrition bias and reporting bias, and all results were reported as previously registered. We considered five studies to be free from other sources of bias [12, 21, 22, 26, 27], while three studies were assessed unclear [23–25]. Assessment result of the risk of bias was shown in Fig 2, and the detailed information was summarized in S2 Table.

### The outcome of effectiveness: Postoperative pain scores

**Fig 3:**
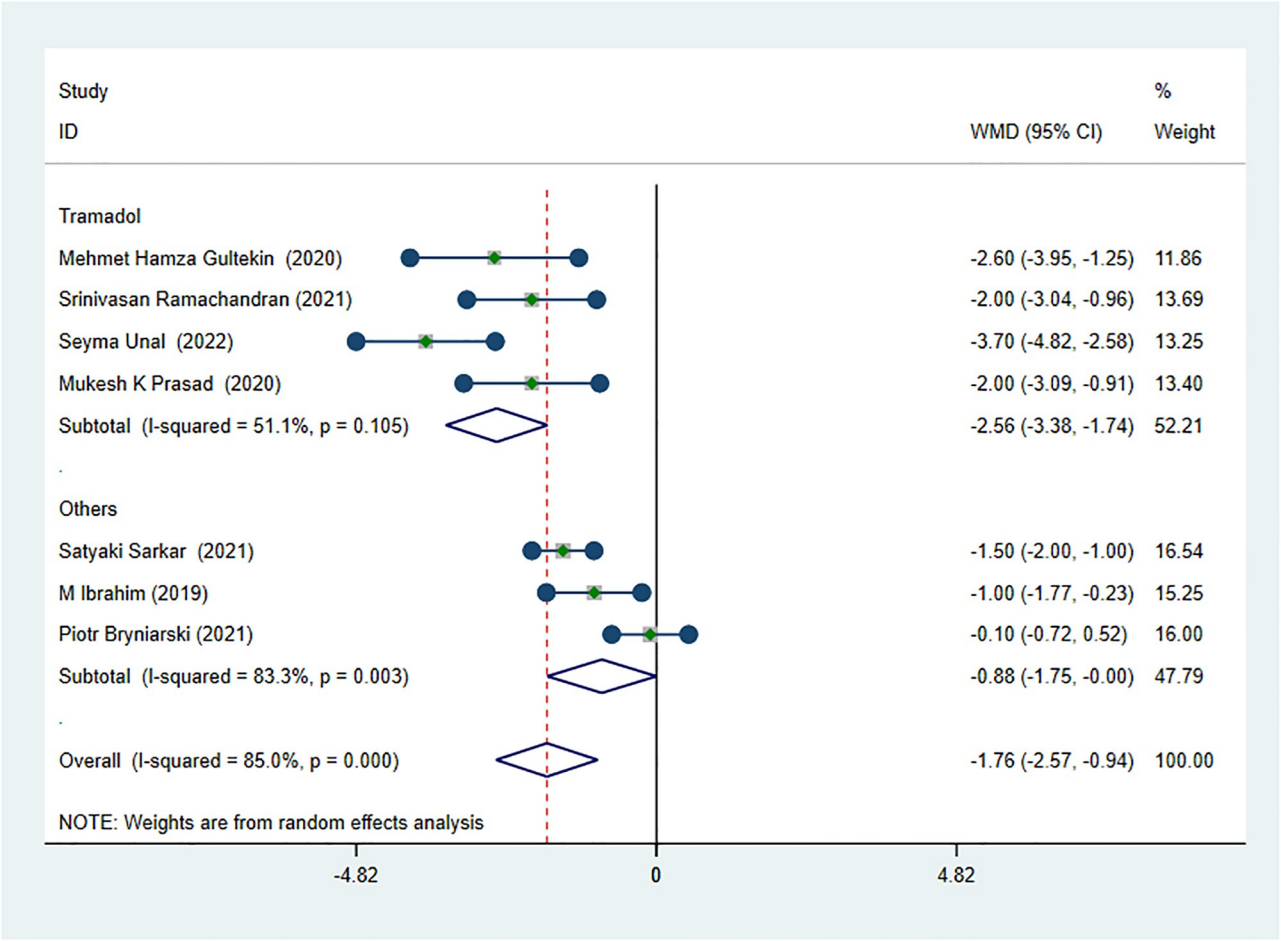
Forest plot displaying the results of pain response score and the results of subgroup analysis by type of postoperative analgesia.

There were 7 studies assessing the efficacy of ESPB in reducing postoperative pain score compared with no block or other blocks [12, 21–26]. According to the finding of meta-analysis, ESPB has a significant effect on improving postoperative pain in patients (MD −1.76; 95% CI −2.57 to −0.94; I ^2^ = 85%; *p*<0.01) (Fig 3).

**Fig 4:**
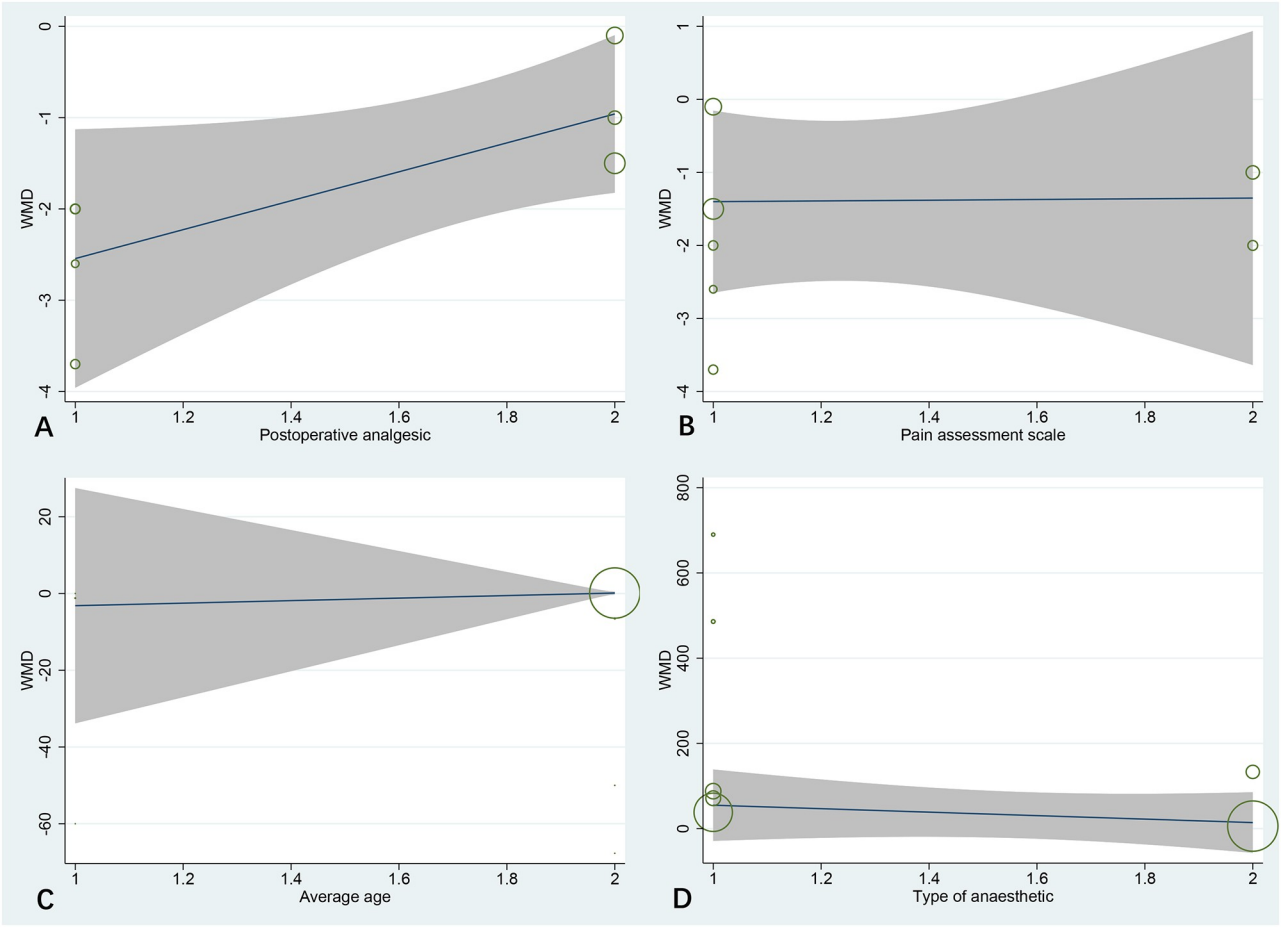
Results of meta-regression analysis.

The result of sensitivity analyses shown that the type of postoperative analgesia may be the main source of the high-heterogeneity between studies. While this result was also confirmed by meta-regression analysis and explained 63.69% of the between-study heterogeneity (*P* = 0.037; 95% CI 1.16 to 24.89; Fig 4A). The subgroup-analysis found that the postoperative analgesia was not significant in patients receiving tramadol. Furthermore, meta-regression excluded the type of pain assessment scale was the reason of high-heterogeneity (*P* = 0.706; Fig 4B).

### The outcome of effectiveness: Postoperative analgesic consumption

**Fig 5:**
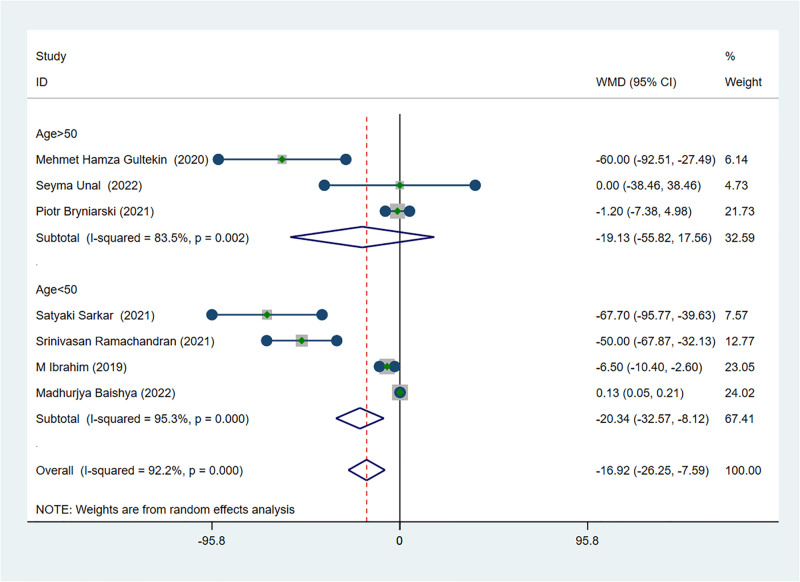
Forest plot displaying the results of postoperative analgesic consumption and the results of subgroup analysis by mean age.

Seven studies provided comparative data on postoperative analgesic consumption [12, 21–25, 27]. The results showed that compared with the control group, patients received ESPB revealed significant advantage in reducing postoperative analgesic consumption (MD −16.92; 95% CI −26.25 to −7.59; I ^2^ = 92.2%; *p*<0.01) (Fig 5). Subsequently, based on the results of subgroup-analysis, we found that the mean age was not the main source of heterogeneity between studies. While this result was also verified by meta-regression (*P* = 0.722; Fig 4C).

### The outcome of effectiveness: The time of first request for postoperative analgesia

**Fig 6:**
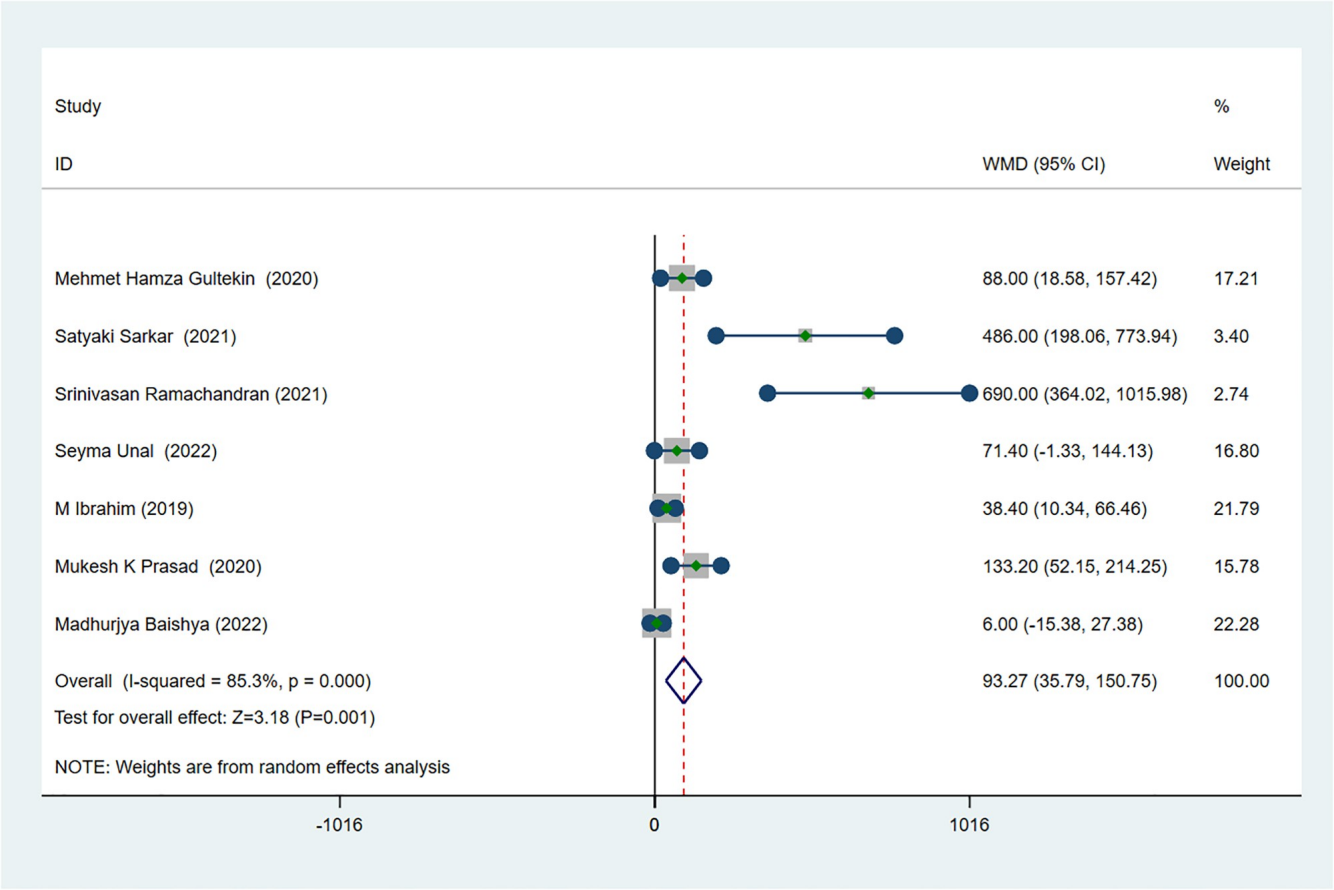
Forest plot displaying the results of the time of first request for postoperative analgesia.

Seven studies reporting the first time in additional analgesia in post-operation [12, 21–24, 26]. The synthesized result revealed that the time to first rescue analgesia in patients received ESPB was higher than the control group, and the difference between the groups was statistically significant (MD 93.27; 95% CI 35.79 to 150.75; I ^2^ = 85.3%; *p* = 0.001) (Fig 6). Furthermore, meta-regression excluded the type of anesthetics was the possible sources of heterogeneity (*P* = 0.468) (Fig 4D).

### The safety of effectiveness: The incidence of PONV

**Fig 7:**
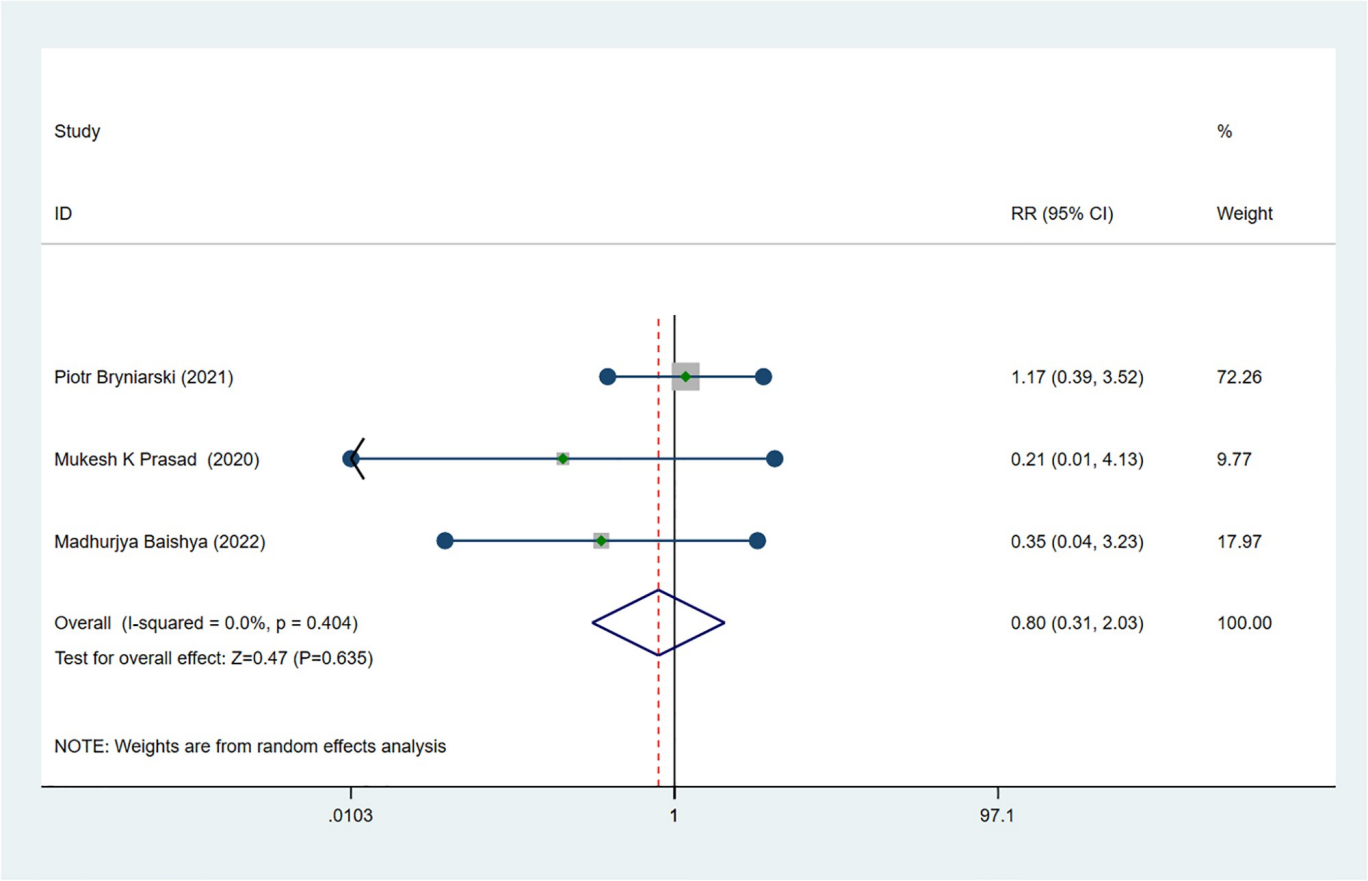
Forest plot displaying the results of incidence of PONV.

Three studies explored the incidence of PONV in ESPB [25–27]. The results showed that no significant difference between ESPB and control groups concerning the incidence of PONV (MD 0.80; 95% CI 0.31 to 2.03; I ^2^ = 0%; *p* = 0.404) (Fig 7).

## Discussion

In order to avoid the impact of publication bias on the research and to provide highest quality evidence to readers [28], this meta-analysis conducted a strict quality control of the included studies and confirmed that the prospective trial registration had been conducted in all RCTs which were included in the final meta-analysis. Our meta-analysis found that ESPB showed significant analgesic advantage regardless of whether the control group received placebo or other anesthesia methods (general anesthesia, local anesthesia and TPVB intrathecal morphine), which was different from previous meta-analysis in which two crucial relevant studies were lacked [29]. Additionally, this meta-analysis not only considered the VAS scale but also analyzed the NRS scale when studying the effect of ESPB on postoperative analgesia for PCNL. The combined results showed strong evidence that ESPB was beneficial for improving postoperative pain response in patients received PCNL. Meta-regression and subgroup analysis was used to further explore the heterogeneity of results in our meta-analysis which was not done in Yucheng Ma’s study. Moreover, the sensitivity analysis did not substantially alter the effect of ESPB on postoperative analgesia of PCNL.

In order to eliminate the influence of natural recovery and early postoperative analgesia and increase the accuracy of meta-analysis results, the pain score at 2h after surgery was considered as the main outcome of effectiveness. A study reported that the effective postoperative analgesia will only occur in the early postoperative period (within 2 hours), if the 35% reduction in pain score was defined as significant [30]. This may be due to the increased consumption of analgesic drugs in the middle and late stages of the control group. However, the long-term analgesic effect of ESPB was not evaluated in our meta-analysis since the limitation of included studies.

For the effectiveness of ESPB, the result of systematic meta-analysis showed that ESPB could significantly decrease the postoperative pain score in patients received PCNL compared with no block or other block. The influence of the type of local anesthetic for this result was not to be excluded since the bupivacaine was selected as local anesthetic in all but one study. However, the final results of meta-analysis were the same whether the study was deleted or not according to the sensitivity analysis. The type of postoperative analgesics might be a major source of heterogeneity after the sensitivity analysis, and this finding was also demonstrated by meta-regression analysis. Furthermore, the possibility that type of pain assessment scale was sources of heterogeneity was excluded by meta-regression. Based on the subgroup-analysis, the result showed that the postoperative analgesic effect of tramadol may not be significant. Moreover, ESPB could effectively reduce the cumulative consumption of analgesics within 24 hours after PCNL, and would not cause obvious postoperative adverse reactions through the result of meta-analysis. According to the result of sensitivity analysis, age greater than 50 might be the source of heterogeneity, but meta-regression analysis excluded this possibility. It is noticeable that based on the result of subgroup-analysis, the postoperative analgesic effect of ESPB was significant regardless of whether the patient was older than 50 years. Additionally, ESPB was also effective in prolonging the time of the first request for postoperative analgesia, and meta-regression analysis ruled out the possibility that the type of anesthetic was the source of heterogeneity. In conclusion, the application of ESPB in postoperative analgesia of PCNL is a safe and effective procedure.

A significant problem in the evaluation of the analgesic effect of ESPB was the absence of a “gold standard”. Some studies suggested that postoperative analgesic consumption could not be used as a reliable measure of analgesic effect [31]. Pain scores may not accurately reflect the postoperative pain response. Because even minor changes in scores may indicate meaningful changes in the pain status of the patient [32, 33]. According to the suggestion of relevant guidelines for pain assessment, in addition to pain scores and analgesic consumption, other measures such as functional performance and a global evaluation of a painful response should be validated in future studies [31, 34, 35]. While the combination of different research tools and measurements is beneficial to a systematic and comprehensive evaluation of the analgesic effect of ESPB.

About the safety of ESPB, many studies have confirmed that ESPB plays a positive role in analgesic effect in pain management after abdominal or chest surgery, and has the advantages of easy implementation and the high block rate [15, 16, 36–40]. A study enrolled with 242 patients reported that there was almost no surgical failure or significant postoperative complications in ESPB [41]. However, it is noticeable that there were only three studies reported the results of PONV in our meta-analysis. More rigorous studies are still needed to further confirm postoperative adverse effects of ESPB in the future.

The ultrasound-guided ESPB has the benefits of accurate anatomical differentiation and ease of operation. Moreover, ESPB is also effective in reducing the risk of postoperative hematoma and peripheral nerve and spinal cord injury, because it is performed away from the spinal cord and is not surrounded by prominent large vessels, nerves and organs [42]. Moreover, the duration of postoperative analgesia can be prolonged by postoperative indwelling catheter.

Presently, the controversy still exists in the mechanism of ESPB. Previous studies through cadaveric and radiological methods have demonstrated that ESPB may exert visceral and somatic analgesic effects similar to epidural anesthesia through local anesthetic diffusion to paravertebral, intercostal and epidural [11, 43–47]. However, the same results were not obtained in a recent cadaver study [48]. Nevertheless, like any other block anesthesia methods, the LAST recognition and management algorithms should be performed especially if high dosage of local anesthetics are utilized [49]. The ideal concentration and dosage of local anesthetics need to be further investigated in the future. Study reported that telemetry monitoring could be conducted to increase the safety of patients if the continuous ESPB is utilized [49]. The drug-induced sleep endoscopy (DISE) is useful in identifying the site, type, and pattern of upper airway obstruction/collapse in patients [50]. Furthermore, ESPB could be combined with multimodal analgesic protocols in future studies [51]. The main findings and clinical implications of this meta-analysis were summarized in S3 Table.

### Limitation

Some limitations were still existing in our study, such as the test of funnel plot symmetry was not conducted since only eight studies were included in this meta-analysis. Trial Sequential Analysis was not used to assess the robustness of the results [52]. Furthermore, this study only considered the articles written in English, which may contain selection bias. Despite our attempts to contact the authors, the postoperative adverse effects other than PONV were not analyzed due to scarce data. The heterogeneous sources of research results were analyzed in our meta-analysis as much as possible, but this does not exclude the possibility of other sources of heterogeneity existing. Although there are several studies which compared ESPB with other anesthesia methods, the further exploration could not be conducted in our study for them since the small number of relevant studies and different methods of anesthesia were utilized.

## Conclusion

All in all, our meta-analysis demonstrated that ESPB was a significantly effective and safe anesthesia for PCNL. Furthermore, ESPB has the advantages of simple operation and fewer postoperative complications, which are worth considering as an option for postoperative pain management of PCNL. Nevertheless, in future clinical practice, it is still necessary to further explore the types and dosages of anesthetics and the long-term analgesic effects of ESPB.

## Supporting information

S1 Table Characteristics of included studies. (DOCX)

S2 Table Bias summary of included studies. (DOCX)

S3 Table Summary of meta-analysis main findings and clinical implications. (DOCX)

S1 Checklist RISMA 2020 checklist. (DOCX)
