# Supplementary material for: The safety and efficacy of ultrasound-guided erector spinae plane block in postoperative analgesic of PCNL: A systematic review and meta-analysis
Source: PLoS One. 2023 Jul 14;18(7):e0288781. doi: 10.1371/journal.pone.0288781 (PMC10348577; doi:10.1371/journal.pone.0288781)
Supplement: S1 Table — (DOCX) [file pone.0288781.s001.docx]

**Table S1. Characteristics of included studies**

| Study | Study design | | Participants | Age (ESPB/other) | Block/control | Anesthetic dose | Duration of surgery | Time to assess pain scores | | Primary outcome | Type of analgesic | |
| --- | --- | --- | --- | --- | --- | --- | --- | --- | --- | --- | --- | --- |
| Turkey  2020  Mehmet Hamza Gultekin | | RCT | 30/30 | 55.1±15.7 /52.6±10.8 | ESPB versus no block | 20 cc of 0.5%  bupivacaine | 103.6±40.4 /105.8±49.3 | At 0, 1, 6, 12, 24,hours postoperatively | VAS scores analgesic consumption Time to first rescue analgesia | | | tramadol |
|  |  |  |  |  |  |  |  |  |  |  |  |  |
|  |  |  |  |  |  |  |  |  |  |  |  |  |
|  |  |  |  |  |  |  |  |  |  |  |  |  |
| India  2021  Satyaki Sarkar | | RCT | 17/17 | 18-60 | ESPB versus no block | 20 ml of 0.25% bupivacaine | — | At 2, 12, 24 hours postoperatively | VAS scores analgesic consumption Time to first rescue analgesia | | | tramadol+ paracetamol |
|  |  |  |  |  |  |  |  |  |  |  |  |  |
|  |  |  |  |  |  |  |  |  |  |  |  |  |
|  |  |  |  |  |  |  |  |  |  |  |  |  |
| India 2021 Srinivasan Ramachandran | | RCT | 33/33 | 46.64±13.5 /44.15±14.5 | ESPB versus subcutaneous infiltration | 20 mL of 0.25% bupivacaine | 77.84±11.04 /79.36±11.7 | At 30 min, 60 min, then hourly for six h, followed by four-hourly up to 24 h | NRS scores analgesic consumption Time to first rescue analgesia | | | tramadol |
|  |  |  |  |  |  |  |  |  |  |  |  |  |
|  |  |  |  |  |  |  |  |  |  |  |  |  |
|  |  |  |  |  |  |  |  |  |  |  |  |  |

( Continued on next page )

| Study | Study design | | Participants | Age (ESPB/other) | Block/control | | Anesthetic dose | | Duration of surgery | Time to assess pain scores | | | Primary outcome | Type of analgesic | |
| --- | --- | --- | --- | --- | --- | --- | --- | --- | --- | --- | --- | --- | --- | --- | --- |
| Turkey 2022 Seyma Unal | | RCT | 29/28 | 53.0±10.4/ 52.0±10.5 | ESPB versus no block | 15 ml of 0.5% bupivacaine | | 140.0 (115.5-170.0) /155.0 (115.5-174.8) | | | At 0, 6, 24 hours postoperatively | VAS scores analgesic consumption Time to first rescue analgesia | | | tramadol |
|  |  |  |  |  |  |  |  |  |  |  |  |  |  |  |  |
|  |  |  |  |  |  |  |  |  |  |  |  |  |  |  |  |
|  |  |  |  |  |  |  |  |  |  |  |  |  |  |  |  |
| Egypt 2019 M Ibrahim | | RCT | 25/25 | 46.1/ 44.8 | ESPB versus no block | 30 mL of 0.25% bupivacaine | | 106.8/100.6 | | | At 2, 4, 6, 12, 24, hours postoperatively | NRS scores analgesic consumption Time to first rescue analgesia | | | morphine |
|  |  |  |  |  |  |  |  |  |  |  |  |  |  |  |  |
|  |  |  |  |  |  |  |  |  |  |  |  |  |  |  |  |
|  |  |  |  |  |  |  |  |  |  |  |  |  |  |  |  |
| Poland 2021 Piotr Bryniarski | | RCT | 34/34 | 55.2±10 /57.4±11,3 | ESPB versus general anesthesia | 20 ml of 0.5% bupivacaine | | 89.9±29.3 /88.2±25.2 | | | At 1, 2, 4, 6, 12, 24, hours postoperatively | VAS scores analgesic consumption PONV | | | nalbuphine |
|  |  |  |  |  |  |  |  |  |  |  |  |  |  |  |  |
|  |  |  |  |  |  |  |  |  |  |  |  |  |  |  |  |

( Continued on next page )

| Study | Study design | | | Participants | Age (ESPB/other) | Block/control | | Anesthetic dose | Duration of surgery | Time to assess pain scores | | Primary outcome | Type of analgesic | |
| --- | --- | --- | --- | --- | --- | --- | --- | --- | --- | --- | --- | --- | --- | --- |
| India 2020 Mukesh K Prasad | | RCT | 31/30 | | 41.03±12.58 /37.37±16.81 | ESPB versus no block | 20 ml of 0.375% ropivacaine | | 115.66±13.25 /110.8±10.83 | | At 1, 2, 3, 4, 6, 12, 18, 24, hours postoperatively | VAS scores analgesic consumption Time to first rescue analgesia PONV | | tramadol |
|  |  |  |  |  |  |  |  |  |  |  |  |  |  |  |
|  |  |  |  |  |  |  |  |  |  |  |  |  |  |  |
| India 2022  Madhurjya Baishya | | RCT | 30/30 | | 36.67±12.76  /40.21±11.96 | ESPB versus TPVB intrathecal morphine | 20 ml of 0.375% ropivacaine | | 122.03±24.50  /127.17±23.24 | | At 0, 0.5, 1, 2, 4, 6, 24, hours postoperatively | VAS scores  analgesic consumption  Time to first rescue analgesia  PONV | | fentanyl |
|  |  |  |  |  |  |  |  |  |  |  |  |  |  |  |
|  |  |  |  |  |  |  |  |  |  |  |  |  |  |  |
|  |  |  |  |  |  |  |  |  |  |  |  |  |  |  |
|  |  |  |  |  |  |  |  |  |  |  |  |  |  |  |
