# Supplementary material for: The safety and efficacy of ultrasound-guided erector spinae plane block in postoperative analgesic of PCNL: A systematic review and meta-analysis
Source: PLoS One. 2023 Jul 14;18(7):e0288781. doi: 10.1371/journal.pone.0288781 (PMC10348577; doi:10.1371/journal.pone.0288781)
Supplement: S2 Table — (DOCX) [file pone.0288781.s002.docx]

| **Table S2. Bias Summary of Included Studies** | | | | | | | |
| --- | --- | --- | --- | --- | --- | --- | --- |
| **Studies** | **Randomization** | **Allocation bias** | **Performance bias** | **Detection bias** | **Attrition bias** | **Reporting bias** | **Other bias** |
| **Mehmet Hamza Gultekin**  **2020** | **Low risk of bias** | **Unclear risk of bias** | **Unclear risk of bias** | **Low risk of bias** | **Low risk of bias** | **Low risk of bias** | **Low risk of bias** |
|  | *"Randomization was performed using a computer‐based list before the operation."*  Randomization of the study was made by blocks that were generated in computer. | No relevant quote found in article. | Insufficient information to permit judgment of ‘Low risk’ or ‘High risk’. | *"All patients and an independent observer who was responsible for follow‐up during 24 postoperative hours were blinded to the randomization groups."*  Blinding of outcome assessment ensured, and unlikely that the blinding could have been broken. | No missing outcome data reported. | All outcomes specified in the methods were reported. | No evidence of other bias. |
| **Satyaki Sarkar**  **2021** | **Low risk of bias** | **Unclear risk of bias** | **Unclear risk of bias** | **Unclear risk of bias** | **Low risk of bias** | **Low risk of bias** | **Low risk of bias** |
|  | The authors randomly divided the participants into two groups. | The method of concealment is not described or not described in sufficient detail to allow a definite judgement. | Insufficient information to permit judgment of ‘Low risk’ or ‘High risk’. | The method of detection bias is not described or not described in sufficient detail to allow a definite judgement. | No missing outcome data reported. | All outcomes specified in the methods were reported. | No evidence of other bias. |
| **Srinivasan Ramachandran 2021** | **Low risk of bias** | **Low risk of bias** | **Unclear risk of bias** | **Low risk of bias** | **Low risk of bias** | **Low risk of bias** | **Low risk of bias** |
|  | *"...with the help of block (created by an external person) randomisation technique..."*  The investigators describe a random component in the sequence generation process. | *"...using a sealed opaque, sequentially numbered envelope for allocation concealment..."*  Participants and investigators enrolling participants could not foresee assignment because one of the following, or an equivalent method, was used to conceal allocation: Sequentially numbered, opaque, sealed envelopes. | No relevant quote found in article. | *"NRS scores were assessed by an independent observer who was blind to the group assignment."*  Blinding of outcome assessment ensured, and unlikely that the blinding could have been broken. | No missing outcome data reported. | All outcomes specified in the methods were reported. | The study appears to be free of other sources of bias. |
| **Seyma Unal**  **2022** | **Low risk of bias** | **Unclear risk of bias** | **Unclear risk of bias** | **Low risk of bias** | **Low risk of bias** | **Low risk of bias** | **Unclear risk of bias** |
|  | *"After creating two sets of 30 unique numbers from 1 to 60 for each group using an internet-based program (www.randomize.org), the patients were randomly allocated to the control or ESPB group."*  The method of random sequence generation is guaranteed. | The method of concealment is not described or not described in sufficient detail to allow a definite judgement. | No relevant quote found in article. | *"The evaluations were done by a blinded observer independent of the study."*  Blinding of outcome assessment ensured, and unlikely that the blinding could have been broken. | Missing outcome data balanced in numbers across intervention groups, with similar reasons for missing data across groups. | All outcomes specified in the methods were reported. | There may be a risk of bias, but insufficient information to assess whether an important risk of bias exist. |
| **M Ibrahim**  **2019** | **Unclear risk of bias** | **Low risk of bias** | **Low risk of bias** | **Low risk of bias** | **Low risk of bias** | **Low risk of bias** | **Unclear risk of bias** |
|  | Insufficient information about the sequence generation process to permit judgement of ‘Low risk’ or ‘High risk’. | *"The allocated intervention was written on a slip of paper then placed in sealed serially numbered and opaque envelopes."*  Participants and investigators enrolling participants could not foresee assignment because one of the following, or an equivalent method, was used to conceal allocation: Sequentially numbered, opaque, sealed envelopes. | *"Blindness to the treatment group involved the surgical and anesthesia teams, operating theater staff, postanesthesia care unit (PACU) and surgical ward nurses."*  The method of blinding participants and personnel was ensured by the author. | *"Collection and data analysis were performed by the anesthesiologist (Elnabtity A) who was blind to which method had been used."*  Blinding of outcome assessment ensured, and unlikely that the blinding could have been broken. | No missing outcome data reported. | This study protocol is registered (ChiCTR 1800017602). All of the study’s prespecified outcomes have been reported. | There may be a risk of bias, but insufficient information to assess whether an important risk of bias exist. |
| **Piotr Bryniarski 2021** | **Low risk of bias** | **Low risk of bias** | **Unclear risk of bias** | **Unclear risk of bias** | **Low risk of bias** | **Low risk of bias** | **Unclear risk of bias** |
|  | *"Random assignment was ensured using a sequence generated by a free resource for researchers"*  Randomization of the study was made by blocks that were generated in computer. | *"Allocation concealment was ensured as the numbers were placed into sealed opaque envelopes and randomly chosen by the anesthetist scheduled to administer anesthesia."*  Participants and investigators enrolling participants could not foresee assignment because one of the following, or an equivalent method, was used to conceal allocation: Sequentially numbered, opaque, sealed envelopes. | The study did not address this outcome. | Insufficient information to permit judgment of ‘Low risk’ or ‘High risk’. | Reasons for missing outcome data unlikely to be related to true outcome. | All outcomes specified in the methods were reported. | There may be a risk of bias, but insufficient information to assess whether an important risk of bias exist. |
| **Mukesh K Prasad 2020** | **Low risk of bias** | **Unclear risk of bias** | **High risk of bias** | **Unclear risk of bias** | **Low risk of bias** | **Low risk of bias** | **Low risk of bias** |
|  | *"surgeries were randomly assigned into two groups using the chit and box method."*  The investigators describe the method of randomization. | Insufficient information to permit judgement of ‘Low risk’ or ‘High risk’ as it remains unclear whether envelopes were sequentially numbered, opaque and sealed. | No blinding or incomplete blinding, and the outcome is likely to be influenced by lack of blinding. | Insufficient information to permit judgment of ‘Low risk’ or ‘High risk’. | No missing outcome data reported. | The trial was registered and all outcomes specified in the methods were reported. | The study appears to be free of other sources of bias. |
| **Madhurjya Baishya 2022** | **Low risk of bias** | **Low risk of bias** | **Unclear risk of bias** | **Low risk of bias** | **Low risk of bias** | **Low risk of bias** | **Low risk of bias** |
|  | *"Patients were randomly allotted to one of the two groups –Group 1: ESPB group and Group 2: IT group – depending on a computer-generated random number table."*  Randomization of the study was made by blocks that were generated in computer. | *"Allocation concealment was achieved by enclosing assignments in sealed, opaque, prenumbered envelopes that were opened only after the patient was brought into the operating theater."*  Participants and investigators enrolling participants could not foresee assignment because one of the following, or an equivalent method, was used to conceal allocation: Sequentially numbered, opaque, sealed envelopes. | Insufficient information to permit judgment of ‘Low risk’ or ‘High risk’. | *"All patients were examined by an independent observer who was unaware of the group assignment."*  Blinding of outcome assessment ensured, and unlikely that the blinding could have been broken. | No missing outcome data reported. | All outcomes specified in the methods were reported. | No evidence of other bias. |

Note: Italics shows direct quotes from articles.
