# Supplementary figures and images for: The safety and efficacy of ultrasound-guided erector spinae plane block in postoperative analgesic of PCNL: A systematic review and meta-analysis
Source: PLoS One. 2023 Jul 14;18(7):e0288781. doi: 10.1371/journal.pone.0288781 (PMC10348577; doi:10.1371/journal.pone.0288781)

**Table S3. Summary of meta-analysis main findings and clinical implications**


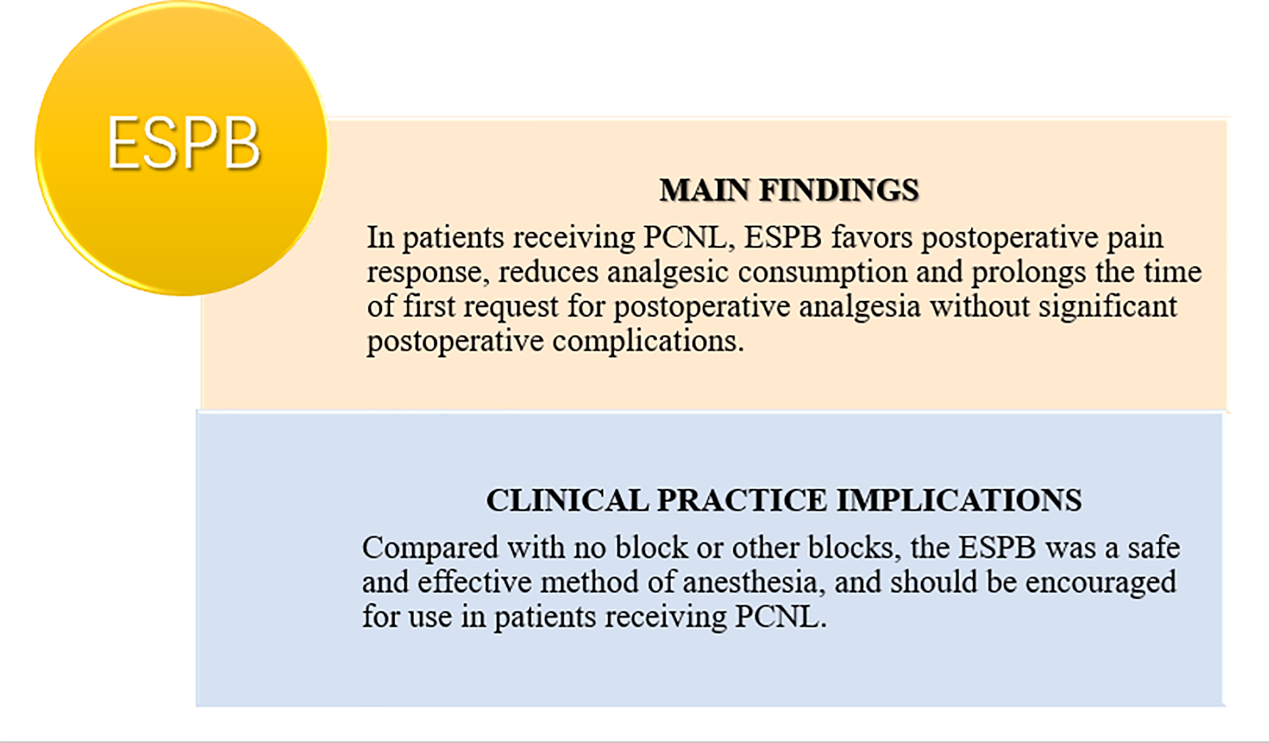

Supplement: S3 Table — (DOCX) [file pone.0288781.s003.docx]
